# Protocol for the isolation of mouse senescence-associated CD4^+^ T cells using flow cytometry and functional assays

**DOI:** 10.1016/j.xpro.2023.102472

**Published:** 2023-07-28

**Authors:** Yuji Fukushima, Nagahiro Minato, Masakazu Hattori

**Affiliations:** 1 Department of Regulation of Neurocognitive Disorders (Cyn-K project), Graduate School of Medicine, Kyoto University, 53 Shogoin-Kawahara-cho, Sakyo-ku, Kyoto 606-8507, Japan; 2 Department of Immunosenescence, Graduate School of Medicine, Kyoto University, 53 Shogoin-Kawahara-cho, Sakyo-ku, Kyoto 606-8507, Japan; 3 Medical Innovation Center, Graduate School of Medicine, Kyoto University, 53 Shogoin-Kawahara-cho, Sakyo-ku, Kyoto 606-8507, Japan; 4 Laboratory of Tumor Tissue Response, Graduate School of Medicine, Kyoto University, 53 Shogoin-Kawahara-cho, Sakyo-ku, Kyoto 606-8507, Japan

**Keywords:** Cell Culture, Cell Isolation, Flow Cytometry/Mass Cytometry, Immunology, Signal Transduction

## Abstract

Senescence-associated (SA) CD4^+^ T cells, which increase with age, may underlie the development of autoimmunity and chronic inflammation, but their pathological function remains understudied. Here, we present a protocol to isolate CD153^+^ SA-T cells and evaluate their characteristic responses upon T cell receptor stimulation. We describe steps for the isolation of CD153^+^ SA-T cells using flow cytometry and *in vitro* culture with stimulatory antibodies against CD3, CD28, and CD153. We then detail the assessment of the proliferation capacity and cytokine production.

For complete details on the use and execution of this protocol, please refer to Fukushima et al. (2022).^1^

## Highlights

• Isolation of CD153^+^ SA-T cells from mouse spleen using flow cytometry
• Stimulation of CD153^+^ SA-T cells with anti-CD3, CD28, and CD153 antibodies
• Analysis of Ki67 expression for assessing the proliferation capacity
• Quantification of osteopontin production by enzyme-linked immunosorbent assay

## Abstract (editor highlights)

Publisher’s note: Undertaking any experimental protocol requires adherence to local institutional guidelines for laboratory safety and ethics.

## Graphical abstract

**Figure:**
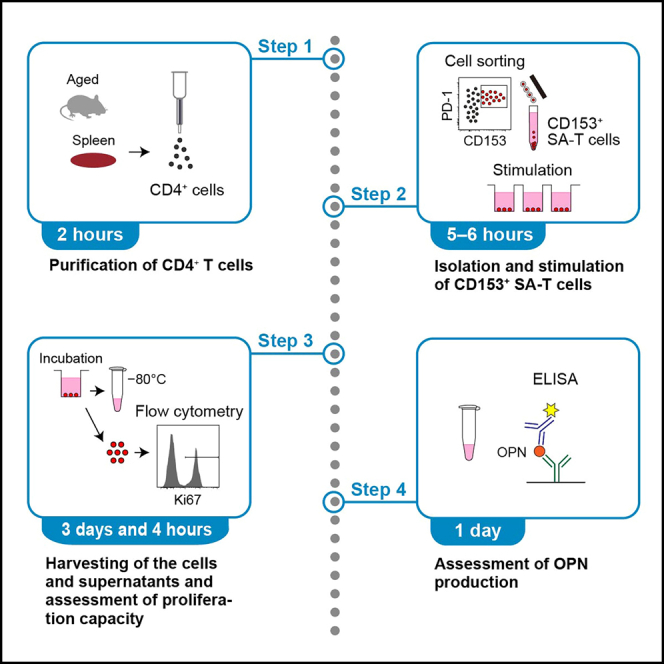

## Before you begin

Senescence-associated (SA) T cells were initially identified as programmed cell death protein 1 (PD-1^+^) CD44^hi^ CD4^+^ T cells.^2^ Recently, however, SA-T cells expressing CD153 (CD153^+^ SA-T cells) have garnered increasing attention because of their potential relationship to the pathogenesis of systemic lupus erythematosus, type 2 diabetes, and chronic kidney disease.^1^^,^^3^^,^^4^^,^^5^^,^^6^^,^^7^ In response to T cell receptor (TCR) stimulation, CD153^+^ SA-T cells have diminished proliferation capacity and produce large amounts of a proinflammatory cytokine osteopontin (OPN), at the cost of typical T cell cytokines such as interleukin 2 (IL-2). Interestingly, concomitant engagement of CD153 enhances TCR signaling and upregulates the proliferation capacity and cytokine production of CD153^+^ SA-T cells.

**Figure 1:**
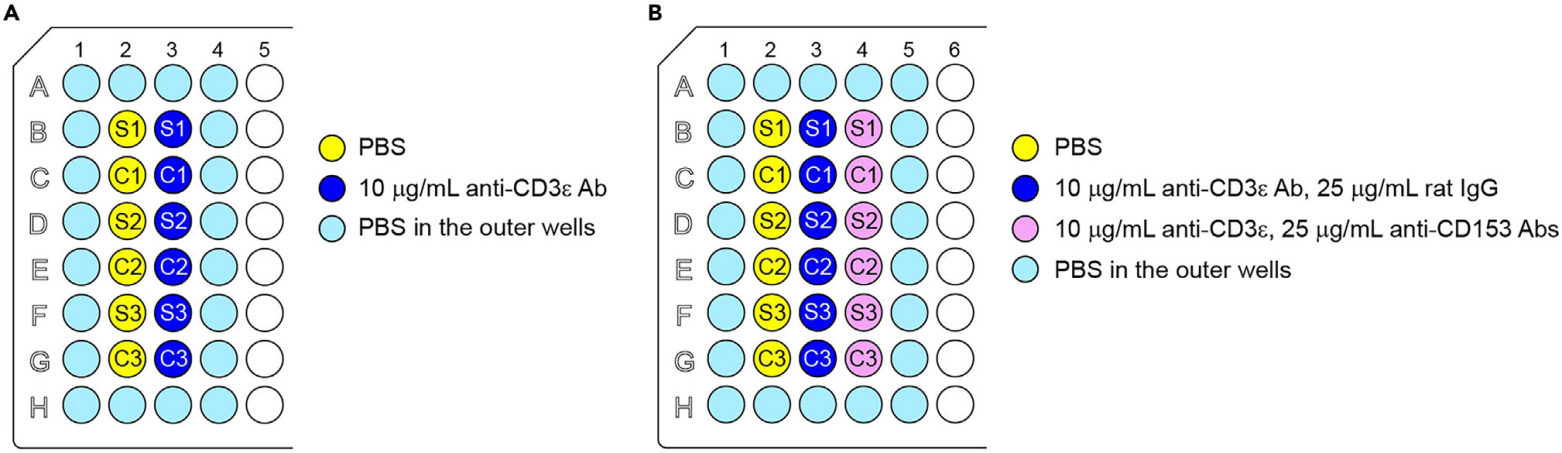
Plate layout for *in vitro* stimulation (A) Example of a 96-well plate layout for stimulation via CD3ε/CD28 (n = 3). S1 and C1 indicate wells for CD153^+^ SA-T cells and PD-1^–^ CD44^hi^ cells, respectively. S2/3 and C2/3 indicate wells for biological replicates of S1 and C1, respectively. (B) Example of a 96-well plate layout for stimulation via CD3ε/CD28/CD153 (n = 3). S1 and C1 indicate wells for enriched CD153^+^ SA-T cells and PD-1^–^ CD44^hi^ (Lag3^–^ CD121b^–^ CD25^–^) cells, respectively. S2/3 and C2/3 indicate wells for biological replicates of S1 and C1, respectively. (A and B) The diluted Abs or buffers and corresponding well positions are shown in the same color.

This protocol describes the isolation and subsequent evaluation of CD153^+^ SA-T cells. Specifically, CD153^+^ SA-T cells were isolated from spleens using a flow cytometry-based method, and the TCR-mediated response was assessed *in vitro* using plate-bound anti-CD3ε (a component of the TCR) and soluble anti-CD28 antibodies (Abs) (Figures 1A and 1B). The additional presence of plate-bound anti-CD153 Ab (clone RM153) served to recapitulate the enhancement of TCR signaling (Figure 1B).

To facilitate the stimulation with plate-bound Abs, anti-CD3ε or anti-CD153 Ab is not used at the isolation step of CD153^+^ SA-T cells. We found that the majority of lymphocyte-activation gene 3 (Lag3^+^) CD121b^–^ CD25^–^ PD-1^+^ CD44^hi^ CD4^+^ T cells were identical to the CD153^+^ SA-T cell fraction on a flow cytometry plot and thus were considered enriched CD153^+^ SA-T cells,^1^ which enabled us to concentrate them without using anti-CD153 Ab. In this protocol, CD153^+^ SA-T cells are first isolated by either method A or B below.

**Figure 2:**
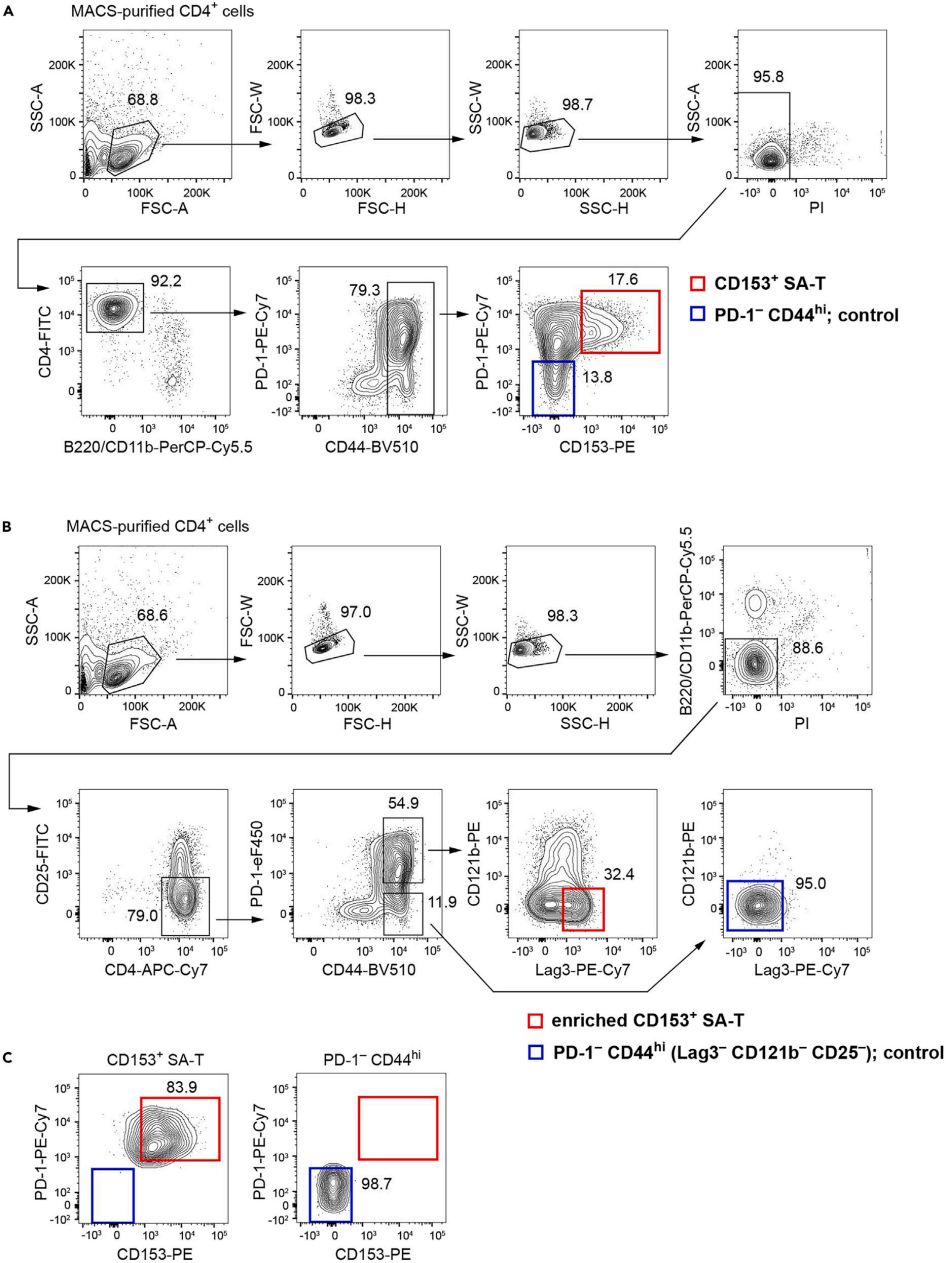
Sorting strategies for CD153^+^ SA-T cells (A) Straight gating strategy. Debris are excluded from the FSC-A/SSC-A plot. Single cells are separated from doublets in plots of FSC-H/FSC-W and SSC-H/SSC-W. PI is used to discriminate dead cells. Although almost all of the cells should be CD4^+^, possible contamination of B220^+^ or CD11b^+^ cells including minor CD4^+^ cells is removed. In a CD44^hi^ cell gate, CD153^+^ SA-T cells (CD153^+^ PD-1^+^) are frequently found. (B) Enrichment gating strategy employed without using anti-CD153 Ab. Lag3^+^ CD121b^–^ CD25^–^ cells in a PD-1^+^ cell fraction concentrates CD153^+^ SA-T cells.^1^ (C) Confirmation of successful sorting. (A–C) A flow cytometer with 488/561/633/405 lasers was used. It should be noted that, unlike this laser system, both PI^+^ dead cells and PerCP-Cy5.5-labeled B220^+^/CD11b^+^ cells were removed together with PerCP-Cy5.5 when 488/633/405/355 lasers were used.
B. Stain the splenic CD4^+^ T cells with staining panel B which lacks both anti-CD3ε and anti-CD153 Abs (Table 2), and isolate “enriched” CD153^+^ SA-T cells with an enrichment gating strategy (Figure 2B).

A. Stain the splenic CD4^+^ T cells with staining panel A which lacks the anti-CD3ε Ab (Table 1), and isolate CD153^+^ SA-T cells with a straight gating strategy (Figure 2A).

**Table 1:** Ab staining panel A to isolate CD153^+^ SA-T cells

Antigen	Clone	Fluorochrome	Final concentration
CD4	GK1.5	FITC	1:200
CD153	RM153	PE	1:200
B220	RA3-6B2	PerCP-Cy5.5	1:200
CD11b	M1/70	PerCP-Cy5.5	1:200
PD-1	J43	PE-Cy7	1:200
CD44	IM7	BV510	1:200

**Table 2:** Ab staining panel B to isolate “enriched” CD153^+^ SA-T cells

Antigen	Clone	Fluorochrome	Final concentration
CD25	PC61.5	FITC	1:200
CD121b	4E2	PE	1:200
B220	RA3-6B2	PerCP-Cy5.5	1:200
CD11b	M1/70	PerCP-Cy5.5	1:200
Lag3	C9B7W	PE-Cy7	1:200
CD4	GK1.5	APC-Cy7	1:200
PD-1	J43	eFluor450	1:200
CD44	IM7	BV510	1:200

Next, stimulate the isolated cells with stimulatory Abs *in vitro*. To examine their response to TCR stimulation (anti-CD3ε/CD28 Abs), the cells isolated by method A (or B) can be used, whereas to examine TCR stimulation with concomitant CD153 engagement (anti-CD3ε/CD28/CD153 Abs), method B should be chosen. Finally, the proliferation capacity and OPN production are assessed by fluorescence-activated cell sorting (FACS) and the enzyme-linked immunosorbent assay (ELISA), respectively. To confirm the characteristic responses of CD153^+^ SA-T cells, it is recommended to isolate and stimulate PD-1^–^ CD44^hi^ CD4^+^ T cells at the same time for comparison.

### Institutional permissions

All animal experiments were approved by the Kyoto University Animal Research Committee and performed according to the Regulations on Animal Experimentation at Kyoto University. Permissions must be obtained from the relevant institutes prior to the animal experiments.

### Mice

Female C57BL/6N mice were purchased from Japan SLC Inc. (Osaka, Japan) and housed in specific pathogen-free conditions in the animal facility at Kyoto University (Kyoto, Japan) for at least 1 week before the experiment. Mice at about 12 months of age were sacrificed.

***Note:*** The mice about ≥12 months of age are an abundant source of CD153^+^ SA-T cells.^1^ However, it should be noted that the frequency of CD153^+^ SA-T cells may be affected by mouse strains, housing conditions, and sex.^3^^,^^8^ For example, frequency of SA-T cells is affected by a genetic predisposition to SLE; lupus prone female NZB/W F_1_ mice shows higher frequency of PD-1^+^ CD44^hi^ CD4^+^ T cells than age-matched non-lupus mice such as male NZB/W F_1_ and female NZW mice.^3^ A number of CD153^+^ SA-T cells are also available using this protocol from female NZB/W F_1_ mice at about 8–10 months of age before mortality. See problem 1 in the troubleshooting section for more detail.

### Coating a cell culture plate with Abs

**Timing: 0.5 h**

1. Dilute the Abs.
  a. For stimulation by anti-CD3ε/CD28 Abs (Figure 1A), dilute the anti-CD3ε Ab in phosphate-buffered saline (PBS) to a final concentration of 10 μg/mL.
  b. For stimulation by anti-CD3ε/CD28/CD153 Abs (Figure 1B), dilute anti-CD3ε and anti-CD153 Abs in PBS to final concentrations of 10 and 25 μg/mL, respectively. Make a negative control of the CD153-mediated effects by diluting anti-CD3ε and rat IgG (instead of anti-CD153 Ab) in PBS to a final concentration of 10 and 25 μg/mL, respectively.

***Note:*** Prepare the Abs in a sterile hood. Anti-CD28 Ab is unnecessary at this step. It is mixed with the cell suspension at a cell culture step.

2. Add 100 μL diluted Abs and PBS (non-stimulation control) to a flat-bottom 96-well plate (Figures 1A and 1B). Add 200 μL PBS to the outer wells. As necessary, make biological replicates.

***Note:*** PBS in the outer wells serves to prevent the coating solution and culture medium from evaporation, which can affect the concentrations of the soluble factors.

3. Wrap the plate with a plastic wrap. Incubate at 4°C for 16–20 h.

## Key resources table

**Table:** 

REAGENT or RESOURCE	SOURCE	IDENTIFIER
**Antibodies**		

Anti-CD4 (GK1.5), FITC (1:200)	Tonbo Biosciences	Cat# 35-0041; RRID: AB_2621665
Anti-CD4 (GK1.5), APC-Cy7 (1:200)	BioLegend	Cat# 100414; RRID: AB_312699
Anti-B220 (RA3-6B2), PerCP-Cy5.5 (1:200)	BioLegend	Cat# 103236; RRID: AB_893354
Anti-CD11b (M1/70), PerCP-Cy5.5 (1:200)	Invitrogen	Cat# 45-0112-82; RRID: AB_953558
Anti-CD25 (PC61.5), FITC (1:200)	Tonbo Biosciences	Cat# 35-0251; RRID: AB_2621685
Anti-CD44 (IM7), BV510 (1:200)	BD Biosciences	Cat# 563114; RRID: AB_2738011
Anti-CD121b (4E2), PE (1:200)	BD Biosciences	Cat# 554450; RRID: AB_395399
Anti-CD153 (RM153), PE (1:200)	Invitrogen	Cat# 12-1531-81; RRID: AB_465883
Anti-Ki67 (16A8), APC (1:200)	BioLegend	Cat# 652405; RRID: AB_2561929
Anti-Lag3 (C9B7W), PE-Cy7 (1:200)	BioLegend	Cat# 125226; RRID: AB_2715764
Anti-PD-1 (J43), PE-Cy7 (1:200)	Invitrogen	Cat# 25-9985-82; RRID: AB_10853805
Anti-PD-1 (J43), eFluor 450 (1:200)	Invitrogen	Cat# 48-9985-82; RRID: AB_2574139
Anti-CD28 (37.51), Ultra-LEAF purified (1:500)	BioLegend	Cat# 102116; RRID: AB_11147170
rat IgG, purified (25 μg/mL for plate coating)	Sigma-Aldrich	Cat# I4131; RRID: AB_1163627
Anti-CD153 (RM153), purified (25 μg/mL for plate coating)	Dr. Hideo Yagita	N/A
Anti-CD3ε (145-2C11), purified (10 μg/mL for plate coating)	In-house	N/A
Anti-CD16/32 (2.4G2), hybridoma supernatant (the optimal dilution should be determined for each lot)	In-house	N/A

**Chemicals, peptides, and recombinant proteins**		

Propidium iodide (PI)	Sigma-Aldrich	Cat# P4170
Fixable Viability Dye eFluor 780 (FVD780)	eBioscience	Cat# 65-0865-14
Fetal bovine serum (FBS)	Biowest	Cat# S1820-500
Bovine serum albumin (BSA)	Nacalai Tesque	Cat# 01863-48
0.5 M EDTA	Nippon Gene	Cat# 311-90075
Phosphate-buffered saline (PBS) (1×)	Nacalai Tesque	Cat# 14249-24
PBS powder	Nissui	Cat# 08192
NH_4_Cl	Nacalai Tesque	Cat# 02424-55
KHCO_3_	Nacalai Tesque	Cat# 28414-85
Na_2_EDTA	Nacalai Tesque	Cat# 09592-45
Penicillin-streptomycin solution	Nacalai Tesque	Cat# 09367-34
β-Mercaptoethanol	Sigma-Aldrich	Cat# M3148
RPMI1640	Gibco	Cat# 11875-093
Trypan blue stain (0.4%)	Gibco	Cat# 15250-061
TMB chromogen solution	Thermo Fisher Scientific	Cat# 002023
1 M H_2_SO_4_	Nacalai Tesque	Cat# 95626-06
Polyoxyethylene sorbitan monolaurate (Tween-20)	Nacalai Tesque	Cat# 35624-15

**Critical commercial assays**		

CD4 (L3T4) Microbeads, mouse	Miltenyi	Cat# 130-117-043
Foxp3/Transcription Factor Staining Buffer Set	eBioscience	Cat# 00-5523-00
Mouse Osteopontin DuoSet ELISA	R&D systems	Cat# DY441

**Experimental models: Organisms/strains**		

Mouse: C57BL/6N (wild-type, female, about 12-month-old)	Japan SLC	C57BL/6NCrSlc

**Software and algorithms**		

FlowJo	Treestar	https://www.flowjo.com/
Microsoft Excel	Microsoft	https://www.microsoft.com/en-us/microsoft-365/excel
GraphPad Prism v9	GraphPad	https://www.graphpad.com/scientificsoftware/prism/

**Other**		

Millex-GV filter unit, 0.22 μm	Merck	Cat# SLGV033RS
Millex-HV filter unit, 0.45 μm	Merck	Cat# SLHV033RS
500 mL bottle top vacuum filter, 0.2 μm	Corning	Cat# 430049
500 mL storage bottles	Corning	Cat# 430282
96-well flat-bottom cell culture plate	Corning	Cat# 3596
96-well round-bottom plate	Thermo Fisher Scientific	Cat# 262162
Petri dish, 60 × 15 mm	Greiner Bio-One	Cat# 628161
70-μm cell strainer	FALCON	Cat# 352350
1 mL syringe	Terumo	Cat# SS-01T
15 mL polypropylene conical tube	Greiner Bio-One	Cat# 188271-013
5 mL polystyrene round-bottom tube with cell-strainer cap, 12 × 75 mm	FALCON	Cat# 352235
5 mL polystyrene round-bottom tube, 12 × 75 mm	FALCON	Cat# 352054
LS column	Miltenyi	Cat# 130-042-401
QuadroMACS Separator	Miltenyi	Cat# 130-090-976
MACS MultiStand	Miltenyi	Cat# 130-042-303
Disposable hemocytometer (4-chambers)	Funakoshi	Cat# 521-10
F96 Maxisorp 96-well Nunc Maxisorp plate	Thermo Fisher Scientific	Cat# 442404
ELISA Seal Plate Film	Excel Scientific	Cat# 3-9134-01
CS&T Research Beads	BD	Cat# 655051
BD FACS Accudrop Beads	BD	Cat# 345249
Wellwash	Thermo Fisher Scientific	Cat# 5165000
Sunrise microplate reader	Tecan	Cat# 16039400
Flow cytometer (488/633/405), analyzer; CantoII	BD	N/A
Flow cytometer (488/561/633/405); AriaIII	BD	N/A

## Materials and equipment

**Table:** Preparation of stock solutions

Reagent	Stock solution concentration	Preparation
anti-CD16/32 Ab (2.4G2 hybridoma culture supernatant)	N/A	Cultivate 2.4G2 hybridoma cells in culture medium (shown below) until the confluent state. Harvest the supernatant and filter through a 0.45 μm filter. Make serial dilutions of the supernatant and determine an optimal dilution for blocking of the Fc receptors. Aliquot and store at 4°C for at least 3 months or −80°C for at least 3 years in a sterile condition.
Propidium iodide (PI)	100 μg/mL	Dissolve 10 mg PI in 10 mL PBS and store in aliquots at −30°C (1 mg/mL stock). Furthermore, dilute the 1 mg/mL stock 1:10 in PBS and store in aliquots at −30°C (100 μg/mL stock). Frozen stocks are stable for at least 5 years. For use, thaw an aliquot of the 100 μg/mL stock and store at 4°C for at least 6 months protected from light.
β-mercaptoethanol	100 mM	Dilute 70 μL β-mercaptoethanol (14.3 M) with 9.93 mL PBS and pass through a 0.22 μm filter. Aliquot and store at −30°C for at least 2 years.
Fetal bovine serum (FBS), heat-inactivated	N/A	Thaw frozen FBS in a 37°C water bath and subsequently heat it in a 56°C water bath for 30 min with frequent agitation. Store at 4°C for up to 2 months.
Rat IgG	2.5 mg/mL	Dissolve 10 mg rat IgG in 4 mL sterile PBS in a sterile hood. Aliquot and store at −30°C for at least 2 years. For use, thaw an aliquot and store at 4°C for at least 6 months.
10× PBS for ELISA wash buffer	10×	Dissolve 96 g PBS powder in 1 L MilliQ. Autoclave and store at 20°C–25°C for at least 2 years.

***Note:*** In this protocol, PI is used for excluding dead cells in FACS analyses of cell surface proteins of viable cells, while FVD780 is used in the analyses of intracellular proteins (such as Ki67) of fixed and permeabilized cells. It should be noted that PI cannot be used in the latter analyses.

**CRITICAL:** β-mercaptoethanol is a hazardous reagent. Wear protective groves and carefully handle it.

**CRITICAL:** The solutions for use in cell culture assays should be handled in a sterile condition.

**Table:** ACK lysis buffer

Reagent	Final concentration	Amount
NH_4_Cl	150 mM	8.02 g
KHCO_3_	10 mM	1.00 g
Na_2_EDTA	100 μM	37.2 mg
MilliQ	N/A	Up to 1 L
**Total**	**N/A**	**1 L**

Adjust pH to 7.2–7.4 with 1 M HCl or 1 M NaOH if it is out of range. Filter the solution through a 0.2 μm filter system (Corning #430049, 430282) and store at 4°C for at least 1 year.

**Table:** Culture medium

Reagent	Final concentration	Amount
FBS, heat-inactivated	10% (v/v)	50 mL
Penicillin-streptomycin solution	1×	5 mL
100 mM β-mercaptoethanol	50 μM	0.25 mL
RPMI1640 medium	N/A	Up to 500 mL
**Total**	**N/A**	**500 mL**

Store at 4°C for up to 3–4 weeks.

**Table:** FACS buffer

Reagent	Final concentration	Amount
FBS, heat-inactivated	2% (v/v)	10 mL
PBS	N/A	Up to 500 mL
**Total**	**N/A**	**500 mL**

Store at 4°C for up to 2 months.

**Table:** MACS buffer

Reagent	Final concentration	Amount
Bovine serum albumin (BSA)	0.5% (w/v)	2.5 g
0.5 M EDTA	2 mM	2 mL
PBS	N/A	Up to 500 mL
**Total**	**N/A**	**500 mL**

Filter the solution through a 0.2 μm filter system (Corning #430049, 430282) and store at 4°C for at least 1 year.

**Table:** ELISA washing buffer

Reagent	Final concentration	Amount
Tween 20	0.05% (v/v)	1 mL
10× PBS	1×	200 mL
MilliQ	N/A	Up to 2 L
**Total**	**N/A**	**2 L**

After adding Tween 20 to PBS, stir sufficiently with a magnetic stir bar. Store at 20°C–25°C for 2 months.

**Table:** 1% BSA-PBS

Reagent	Final concentration	Amount
BSA	1% (w/v)	5.0 g
PBS	N/A	Up to 500 mL
**Total**	**N/A**	**500 mL**

Filter the solution through a 0.2 μm filter system (Corning #430049, 430282) and store at 4°C for at least 1 year (in a sterile condition).

### Scissors and tweezers

Autoclave the scissors and tweezers and dry.

## Step-by-step method details

### Purification of CD4^+^ T cells

**Timing: 2 h**

CD4^+^ T cells are purified from the spleens of aged mice with a magnetic cell separation system.

1. Sacrifice two mice by CO_2_ asphyxiation and spray them entirely with alcohol disinfectant.

***Note:*** If three biological replicates (n = 3) have been prepared, use a total of six mice; two mice for each pooled sample. Of course, the number of mice per pool should be changed according to the number of CD153^+^ SA-T cells needed. See also Expected Outcomes.

2. Collect the spleens.
  a. In a sterile hood, collect the spleens in a 70 μm cell strainer on a 6 cm petri dish filled with 5 mL FACS buffer.
  b. Mash the spleens with the plunger end of a sterile 1 mL syringe. Grasp the cell strainer using a tweezer and wash the suspension in the strainer with an additional 5 mL FACS buffer.
  c. Transfer the total 10 mL of suspension to a 15 mL conical tube and centrifuge at 350 × *g* for 5 min at 4°C.
  d. Aspirate the supernatant.

**CRITICAL:** All steps from tissue collection to cell culture must be performed under sterile conditions.

3. Lyse the red blood cells.
  a. Briefly dissociate the cell pellet by tapping the tube and suspend the cells thoroughly in 2 mL ACK lysis buffer.
  b. Incubate for 2 min at 20°C–25°C.
  c. Add 10 mL FACS buffer, mix by inversion and centrifuge at 350 × *g* for 5 min at 4°C.
  d. Aspirate the supernatant.
4. Block the Fc receptors.
  a. Suspend the cells in 5 mL anti-CD16/32 Ab (diluted 2.4G2 hybridoma culture supernatant).
  b. Incubate for 15 min at 4°C.
  c. Centrifuge at 350 × *g* for 5 min at 4°C and aspirate the supernatant.

***Optional:*** Count the cells using a hemocytometer. For reference, 0.5–1 × 10^8^ cells are typically obtained from an adult mouse.

***Alternatives:*** Although we have prepared 2.4G2 in-house, Abs blocking Fc receptors are commercially available.

5. Label CD4^+^ T cells with anti-CD4 magnetic beads.
  a. Dilute 200 μL CD4 MicroBeads with MACS buffer to a final volume of 2 mL.
  b. Suspend the cells with the diluted beads and incubate for 30 min at 4°C.
  c. Add 10 mL MACS buffer, mix by inversion and centrifuge at 350 × *g* for 5 min at 4°C.
  d. Aspirate the supernatant and suspend the cells in 1 mL MACS buffer.

***Alternatives:*** Instead of CD4 MicroBeads, a purification kit for unlabeled CD4^+^ T cells is also useful (e.g., mouse CD4^+^ T Cell Isolation Kit; Miltenyi #130-104-454).

6. Purify CD4^+^ T cells by magnetic cell separation.
  a. According to the manufacturer’s instruction, place an LS column (Miltenyi) in the magnetic field of the MACS separator and rinse the column with 3 mL MACS buffer.
  b. Transfer the cell suspension to the column.
  c. Wash the column twice with 3 mL MACS buffer.
  d. Remove the column from the magnet and place on a 15 mL conical tube. Flush out the labeled CD4^+^ cells with 5 mL MACS buffer.
  e. Centrifuge at 350 × *g* for 5 min at 4°C and aspirate the supernatant.

***Optional:*** After flushing, count the cells using a hemocytometer. A rough estimation is that the total cell number will be 1.5–3 × 10^7^ cells per two spleens.

***Note:*** The frequency of CD4^+^ T cells in splenic white blood cells decreases with age. We found that the mean frequency ± standard error of the mean (SEM) was 16.74% ± 0.52% in mice at 43–50 weeks of age (n = 8 mice) in contrast to 23.78% ± 0.89% at 9 weeks of age (n = 5 mice).

***Note:*** This purification step of CD4^+^ T cells allows the isolation of CD153^+^ SA-T cells in a shorter time during a cell sorting step. CD4 MicroBeads do not interfere with anti-CD4 Ab (clone GK1.5) in the staining panels (Tables 1 and 2) and thus are compatible.

### Isolation and stimulation of CD153^+^ SA-T cells

**Timing: 5**–**6 h**

CD153^+^ SA-T cells are isolated using a flow cytometer and stimulated *in vitro*.

7. Stain the cells with Abs.
  a. Suspend the cells in 300 μL of either staining panel A or B containing all required Abs (1:200 dilution with FACS buffer; Tables 1 and 2).
  b. Incubate for 20 min in the dark at 4°C.
  c. Add 10 mL FACS buffer, mix by inversion and centrifuge at 350 × *g* for 5 min at 4°C.
  d. Aspirate the supernatant and suspend the cells in 1–2 mL FACS buffer.
  e. Filter the cell suspension through a 35 μm strainer cap into a 5 mL polystyrene tube and keep on ice.

***Note:*** Although a relatively large number of cells are stained with only 300 μL Abs (approximately 0.5–1 × 10^8^ cells/mL) to avoid rapid consumption of the staining Abs, these cells are stained adequately without any problems for this purpose.

***Note:*** Anti-B220 and CD11b Abs are added to remove possible contaminants of B cells, myeloid cells and plasmacytoid dendritic cells.

8. Set up a flow cytometer according to the instructions of the manufacturer (e.g., AriaIII) and institution. Check the cytometer performance with CS&T beads (BD). Determine the drop delay using Accudrop beads (BD). Use a 70 or 85 mm nozzle for sorting.

***Note:*** We have used flow cytometers with 488/561/633/405 (AriaIII), 488/640/405/355 (AriaII) or 488/561/640/405/355 lasers (Fusion). See also Figure 2 legend.

9. Prepare 10 mL culture medium into 15 mL conical tubes for collection. Coat the whole surface inside the tube with the medium to minimize the attachment of sorted cells on to the surface.
10. Place the 15 mL conical tubes on the two-way 15 mL collection device.
11. Immediately before loading, add a 1/200 volume of 100 μg/mL PI to the sample tube and vortex it to stain the dead cells.
12. Isolate CD153^+^ SA-T cells using strategy a or b.
  a. Straight gating strategy.
    i. Make the plots and gates as shown in Figure 2A.
    ii. Load the sample tube containing the cells stained with panel A.
    iii. Sort CD153^+^ SA-T cells (CD153^+^ PD-1^+^ CD44^hi^ CD4^+^ B220^–^ CD11b^–^) into a collection tube. Simultaneously, sort PD-1^–^ CD44^hi^ cells (CD153^–^ PD-1^–^ CD44^hi^ CD4^+^ B220^–^ CD11b^–^) into another collection tube.
  b. Enrichment gating strategy.
    i. Make the plots and gates as shown in Figure 2B.
    ii. Load the sample tube containing the cells stained with panel B.
    iii. Sort the enriched CD153^+^ SA-T cells (Lag3^+^ CD121b^–^ CD25^–^ PD-1^+^ CD44^hi^ CD4^+^ B220^–^ CD11b^–^) into a collection tube. Simultaneously, sort PD-1^–^ CD44^hi^ (Lag3^–^ CD121b^–^ CD25^–^) cells (PD-1^–^ CD44^hi^ Lag3^–^ CD121b^–^ CD25^–^ CD4^+^ B220^–^ CD11b^–^) into another collection tube.

***Note:*** Set the sample temperature control at 4°C. A high temperature may cause the downregulation of Ab-labeled surface antigens.

***Note:*** Sort a sufficient number of the cells (if possible, at least 1.5 times more cells than the actual number you need).

13. During or at the end of sorting, confirm the successful sorting.
  a. Unload the sample tube.
  b. Mix the collecting medium well with a sterile 1000 μL tip and add a small aliquot (e.g., 200 μL) to a 5 mL polystyrene tube.
  c. Reload the aliquot and confirm that almost all of the cells are plotted within or close to the target gate (Figure 2C).
14. Take a note of the “total sort count” displayed on the Sort Layout Window.
15. Bring the Ab-coated culture plate at 4°C to room temperature (20°C–25°C).
16. Centrifuge the sorted cells.
  a. Centrifuge the sorted cells at 350 × *g* for 15 min at 4°C.
  b. Aspirate the supernatant.
  c. Repeat a for 5 min and b to further remove the supernatant.
  d. Suspend the cells in culture medium to an estimated concentration of 2 × 10^6^ cells/mL based on the “total sort count” (e.g., use 300 μL medium for 6 × 10^5^ counts).

***Note:*** After the first centrifuge and aspiration step (16a, b), a small but non-negligible volume of the supernatant will still remain on the surface of the tube. The second step (16c) allows almost complete removal of the supernatant and then makes the adjustment of the cell concentration accurate at step 18.

17. Count the actual cell number.
  a. Take 10 μL of cell suspension and mix it with 10 μL Trypan Blue Stain (0.4%) by pipetting. Inject 10 μL of the mixed cell suspension into a hemocytometer.
  b. According to the manual, count the unstained alive cells on a microscope and calculate the actual concentration of the cells.

***Note:*** Almost all of the cells should be alive at this point. The actual concentration is usually somehow lower than the estimation due to a possible loss (e.g., attachment of the cells to the collection tube). See problem 2 and 3 in the troubleshooting section for more detail.

18. Adjust the concentration to 1 × 10^6^ cells/mL by adding culture medium.
19. Initiate the stimulation with Abs.
  a. Dilute anti-CD28 Ab 1:250 in culture medium to make a 2× (4 μg/mL) anti-CD28 Ab solution.
  b. Using a 10 μL tip, aspirate the coating solution and PBS from the Ab-coated plate.
  c. Add 50 μL of 2× anti-CD28 Ab solution and culture medium to the corresponding wells (the former to the wells in blue and magenta colors and the latter to the wells in yellow color in Figures 1A and 1B).
  d. Add 50 μL of the cell suspension to the corresponding wells; now each well contains 100 μL.

***Note:*** Aspiration should be performed from the edge of the well. There is no need to mix by pipetting after adding the cell suspension.

20. Incubate the plate at 37°C in a humidified 5% CO_2_ incubator.

***Note:*** As necessary, use the remaining cells for other assays such as cytokine FACS, *in vitro* survival assays, and RNA extraction.

### Harvesting of the cells and supernatants and assessment of proliferation capacity

**Timing: 3 days for incubation and 4 h for assessment**

The proliferation capacity is assessed by FACS.

21. After a 72-h incubation, harvest the cells and supernatant.
  a. Suspend the cultured cells by gentle pipetting using a multichannel pipette and transfer them to a 96-well round bottom plate (the lid of the culture plate is reused in following steps).
  b. Centrifuge the plate at 400 × *g* for 5 min.
  c. Collect 70–80 μL/well of the supernatant and store it in aliquots at −80°C (go to step 35).
  d. Briefly vortex the remaining cells on the bottom of the plate to dissociate the pellets.
  e. Add 100 μL FACS buffer to the wells.

***Note:*** It is highly recommended to observe the cells under a microscope before harvesting. T cell activation is manifested by enlargement in cell size and apparent cell growth.

***Note:*** The supernatant should be stored in aliquots to avoid freeze-thaw cycles. Vortexing the plate should be performed carefully to prevent the cells from scattering away from the wells.

***Alternatives:*** Although the proliferation capacity is assessed by the expression of Ki67, a marker of cell cycle progression, it is also useful to evaluate cell growth with a commercially available kit using an aliquot of the sample.

22. Wash the cells with FACS buffer.
  a. Centrifuge the plate at 400 × *g* for 2 min and discard the supernatant by quickly inverting the plate.
  b. Briefly vortex the plate.
  c. Add 100 μL FACS buffer.
  d. Repeat the steps a and b.
23. Stain the dead cells.
  a. Add 50 μL FVD780 (1:1,000 dilution with FACS buffer).
  b. Incubate the plate for 20 min at 4°C in the dark.

***Note:*** Fluorescence of FVD780 can be read through a filter for Allophycocyanin-Cyanine7 (APC-Cy7) on flow cytometry. Although panel B includes APC-Cy7-labeled anti-CD4 Ab, the fluorescence from the remaining anti-CD4 Ab is far weaker than that from FVD780-stained dead cells at this point and thus cause no problem in excluding dead cells in this flow cytometry assay.

24. Wash the cells with FACS buffer as in step 22.
25. Fix and permeabilize the cells.
  a. Add 50 μL Fixation/Permeabilization buffer to the wells and gently vortex for 10 s.
  b. Incubate the plate for 30 min at 4°C in the dark.

***Note:*** Prior to use, the Fixation/Permeabilization buffer is prepared by mixing 1 volume of Concentrate with 3 volumes of Diluent (included in Foxp3/Transcription Factor Staining Buffer Set).

26. Wash the cells with 1× Permeabilization buffer (1:10 dilution with distilled water) (included in the Foxp3/Transcription Factor Staining Buffer Set).
  a. Centrifuge the plate at 400 × *g* for 2 min and discard the supernatant.
  b. Briefly vortex the plate.
  c. Add 100 μL of 1× Permeabilization buffer.
  d. Repeat steps a and b.
27. Stain intracellular Ki67.
  a. Add 50 μL APC-labeled anti-Ki67 Ab (1:200 dilution with 1× Permeabilization buffer).
  b. Incubate the plate for 30 min at 20°C–25°C in the dark.

***Alternatives:*** PE-labeled anti-Ki67 Ab (BioLegend #652403) is also useful to stain the enriched CD153^+^ SA-T cells (panel B-stained), showing clear separation of Ki67^+^ cells from Ki67^-^ cells.^1^

**Figure 3:**
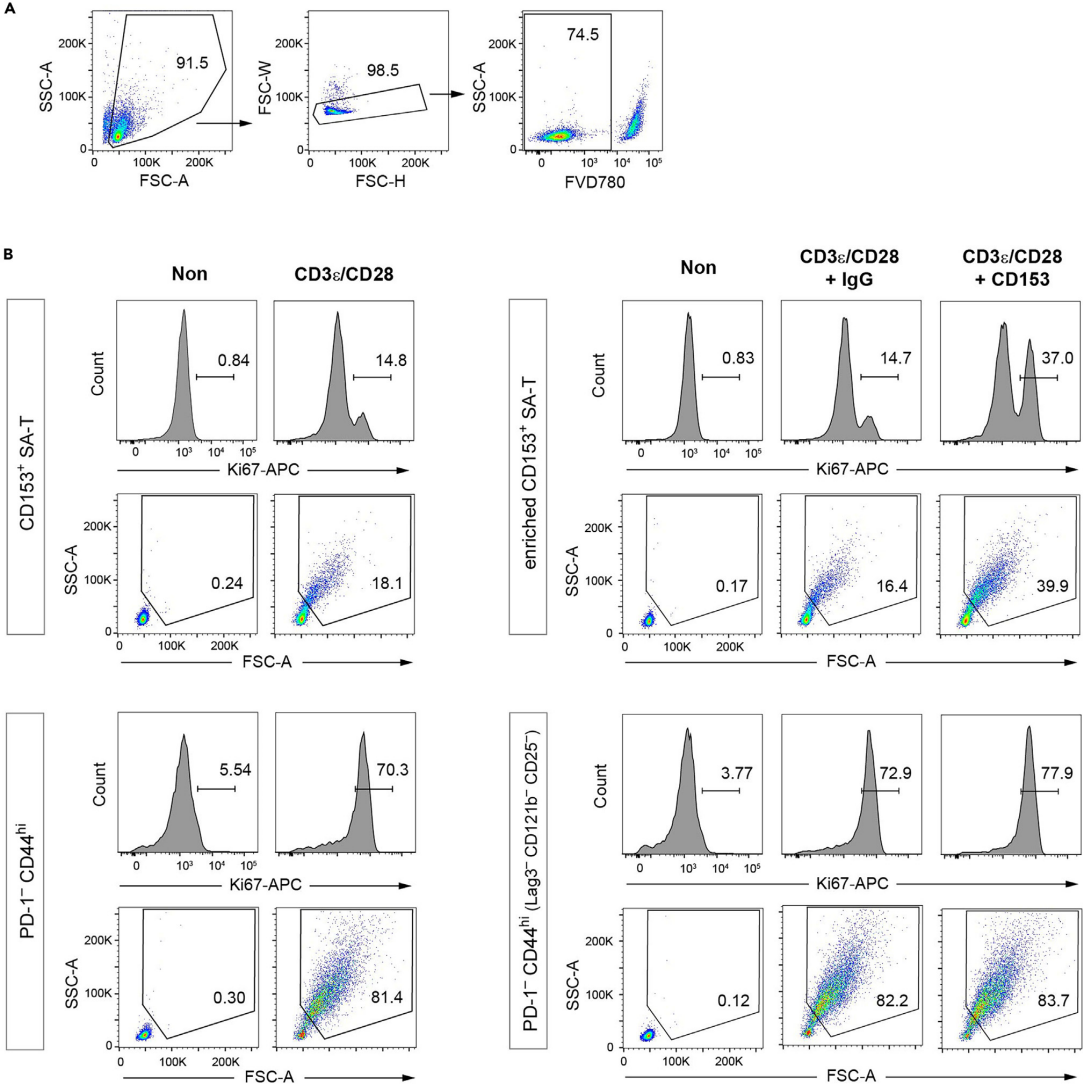
FACS analysis of Ki67 expression (A) Gating for excluding doublet and dead cells. Debris are excluded from the FSC-A/SSC-A plot. Single cells are briefly separated from doublets in plots of FSC-H/FSC-W. FVD780 is used to discriminate dead cells. (B) Ki67 expression and cell size. Flow cytometry histograms depicting the frequency of Ki67^+^ cells and FSC-A/SSC-A plots indicating the frequency of enlarged cells in the FVD780^–^ cell gate are shown. Stimulatory Abs and cell populations (left, cells sorted by the straight gating strategy; right, those by the enrichment gating strategy) are indicated.

***Note:*** Unlike FACS analysis of the cell surface antigens on viable cells, do not add PI.

28. Wash the cells with 1× Permeabilization buffer as in step 26.
29. Wash the cells with FACS buffer as in step 22.
30. Suspend the cells in 150 μL FACS buffer and filter them through a 37 μm nylon mesh into a 5 mL polystyrene tube.
31. Load the samples on the CantoII flow cytometer and assess the frequency of Ki67^+^ cells (Figures 3A and 3B). See also problems 4 and 5 in the troubleshooting section.

### Assessment of OPN production

**Timing: 1 day (∼24 h)**

OPN production is assessed by ELISA using a commercially available kit (R&D).

**Pause point:** The supernatant can be stored at −80°C for at least 2 weeks and thus the experiment can be paused at this point.

32. Coat a 96-well Nunc Maxisorp plate with an anti-OPN capture Ab (included in the kit).
  a. Add 50 μL/well of a working dilution of the capture Ab to the plate.
  b. Seal the plate with a plate seal and incubate it for 16–20 h at 20°C–25°C.

***Note:*** Prepare the wells for samples and recombinant mouse (rm) OPN standards (included in the kit) in duplicate.

***Note:*** Please see the manufacturer’s instruction for details. Of course, you can assess the production of the other cytokines and chemokines using the other kits.

33. Wash the plate with 400 μL/well of ELISA washing buffer three times and then remove the remaining buffer by blotting the inverted plate against paper towels.

***Note:*** We used Wellwash Microplate Washer. It is recommended that a multichannel pipette be used for the following steps to accomplish them quickly.

34. Block the plate.
  a. Add 300 μL/well of 1% BSA-PBS.
  b. Incubating for 1 h at 20°C–25°C.
35. During blocking, prepare the samples and standards.
  a. After dissolving, dilute supernatant samples with 1% BSA-PBS; prepare 110 μL or more for each.
  b. Make 2-fold serial dilutions of rmOPN with 1% BSA-PBS according to the manufacturer’s instructions.

***Note:*** Dilution at 1:100–1:400 is usually appropriate for the supernatant of the cells stimulated by anti-CD3ε/CD28 or anti-CD3ε/CD28/CD153 Abs. Less dilution (e.g., 1:5) is more appropriate for that of non-stimulated cells.

36. Wash the plate as in step 33.
37. Add 50 μL/well of the samples and standards and cover the plate with a plate seal. Incubate for 2 h at 20°C–25°C.
38. Wash the plate as in step 33.
39. Add 50 μL/well of a working dilution of the biotinylated detection Ab (included in the kit) and cover the plate with a plate seal. Incubate it for 2 h at 20°C–25°C.
40. Wash the plate four times as in step 33.
41. Add 50 μL/well of a working dilution of horseradish peroxidase-streptavidin (included in the kit) and cover the plate with a plate seal. Incubate it for 20 min in the dark at 20°C–25°C.
42. Wash the plate five times as in step 33.
43. Add 50 μL/well of TMB solution and cover the plate with a plate seal. Incubate it for 20 min or less in the dark at 20°C–25°C.

***Note:*** Prior to use, bring the TMB solution to room temperature (20°C–25°C).

44. Add 50 μL/well of 1 M H_2_SO_4_ and mix it with a gentle tapping to stop the reaction.

**CRITICAL:** Wear protective groves and carefully use H_2_SO_4_ as it is caustic.

45. Measure the optical density at 450 nm using a microplate reader such as the Sunrise Microplate Reader. For correction, subtract the readings at 620 nm from those at 450 nm. Average the duplicate readings for each sample and standard. Make a standard curve and calculate the concentration of OPN in the samples. See also problem 4 and 5 in the troubleshooting section.

## Expected outcomes

According to the isolation protocol here, we obtained about 7.5 × 10^5^ cells of CD153^+^ SA-T cells from two spleens of mice at about 12 months of age. Note that because CD44^hi^ CD4^+^ T cells express gradually varying levels of PD-1 and CD153 (Figures 2A and 2B), the number available is affected by how strictly CD153^+^ PD-1^+^ cells is gated on a flow cytometry plot.

The FACS analyses of Ki67 (Figures 3A and 3B) showed that 9.94% ± 3.10% of CD153^+^ SA-T cells expressed Ki67 upon stimulation by anti-CD3ε/CD28 Abs in contrast to more than 70% of PD-1^–^ CD44^hi^ cells.^1^ By anti-CD3ε/CD28/CD153 Abs, the frequency of Ki67^+^ in CD153^+^ SA-T cells was increased to 33.34% ± 6.25%.^1^ Besides Ki67 expression, T cell activation was manifested by an enlargement in cell size, which was also confirmed in the same FACS analyses (Figures 3A and 3B).

CD153^+^ SA-T cells produced 16.2 ± 3.13 ng/mL OPN upon stimulation by anti-CD3ε/CD28 Abs and 52.7 ± 2.68 ng/mL by anti-CD3ε/CD28/CD153 Abs.^1^ PD-1^–^ CD44^hi^ cells produced less than 2.5 ng/mL upon Ab stimulation.^1^

## Limitations

This protocol isolates CD153^+^ SA-T cells from the spleens based on their constitutive expression of PD-1 and CD153. However, these surface antigens can be transiently induced *in vitro* upon stimulation,^9^^,^^10^ suggesting that the activated T cells transiently expressing these antigens may also be present *in vivo* and such cells would be indistinguishable from authentic CD153^+^ SA-T cells in this protocol.

An intimate interaction between T cells and antigen-presenting cells allow the appropriate antigen recognition and a subsequent T cell response. A TCR complex consisting of variable TCRαβ and invariable CD3 chains (γ, δ, ε, and ζ) recognizes the antigen presented on a major histocompatibility complex and evokes signaling cascades to activate T cells. The other signaling cascades downstream of CD28 serves to ensure the TCR-mediated response.^11^ Additionally, a number of molecules including CD153, immune checkpoint receptors, and even unrevealed ones may participate in the regulation of TCR signaling. Stimulation by plate-bound anti-CD3ε with soluble anti-CD28 Abs is very useful way to evoke TCR/CD28 signaling *in vitro* and is employed for investigating T cell function. However, it is worth noting that this protocol is simplified compared to the possible complexity of the molecular interactions *in vivo* and thus may not be fully identical. In a similar context, although plate-bound anti-CD153 Ab recapitulates the enhanced TCR-mediated activation of CD153^+^ SA-T cells,^1^ whether it entirely recapitulates the activation mechanism needs to be considered.

Besides the stimulatory Abs, the mixed lymphocyte reaction (MLR) may allow evaluation of the response of CD153^+^ SA-T cells to antigen-presenting cells. CD153^+^ SA-T cells cultured in direct contact with B cells, both of which were isolated from lupus-prone mice, produced more OPN compared to those without B cells.^3^ However, although a minor portion of potential antigen-presenting cells (spontaneous germinal center B cells and age-associated B cells) for CD153^+^ SA-T cells detectably expressed CD30 (a receptor for CD153), the expression levels of the surface protein were apparently weak probably due to progressive shedding of the extracellular domain of CD30,^1^^,^^7^ which may compromise the effects of CD153 on SA-T cells in MLR.

## Troubleshooting

### Problem 1

Frequency of CD153^+^ SA-T cells appears to be low or high compared with that in the literature.

### Potential solution

Note that the frequency may be affected by mouse strains, housing conditions, and sex and that aged mice may occasionally exhibit considerable interindividual difference in the number of CD4^+^ T cell subpopulations. If necessary, assess the basal frequency in the experimental settings using a sufficient number of mice. If it is still too low, check the compensation of your data. For example, be careful of compensation between PE-labeled CD153 and PI because incorrect compensation may push the CD153^+^ cells away from a PI^–^ viable cell gate.

### Problem 2

Dead cells are frequently found after cell sorting.

### Potential solution

Check the gating strategy in which dead cells should be excluded on the basis of PI fluorescence. Prepare new buffers and culture medium. During the purification and isolation steps, the cells should be kept at 4°C or on ice.

### Problem 3

The yield of CD153^+^ SA-T cells after cell sorting is low.

### Potential solution

Confirm that you have used a “polypropylene” 15 mL tube for collection, and coat the whole surface inside the tube with medium before sorting. Centrifuge the sorted cells for a longer time (e.g., 15 min) than usual. Nevertheless, the number of the sorted cells will probably decrease during the following procedures. A simple solution to increase the yield is to use more mice and pool their spleens.

### Problem 4

Unexpectedly, CD153^+^ SA-T cells appear to proliferate substantially and secrete typical T cell cytokines rather than OPN in response to anti-CD3ε and anti-CD28 Abs.

### Potential solution

Carefully confirm whether the compensation is adjusted correctly. Incorrect compensation may push the PD-1^–^ cells into a PD-1^+^ cell fraction. Considering the primary finding that CD153^+^ SA-T cells are increased with age, it is preferable to isolate the accumulated CD153^+^ SA-T cells from mice at or after middle age (≥12 months) rather than collect a small number of cells from younger mice; this will reduce the likelihood of heterogeneity caused by transiently activated cells (see Limitations).

### Problem 5

Additional presence of plate-coated anti-CD153 Ab does not enhance Ki67 expression and OPN production of enriched CD153^+^ SA-T cells upon TCR stimulation.

### Potential solution

Ensure successful enrichment by staining an aliquot of the sorted cells with both PE-labeled anti-CD153 Ab and isotype-matched control IgG. Successful enrichment will manifest as about 75% purity of CD153^+^ cells. For plate coating, use anti-CD153 Ab clone RM153, since the epitope may also be responsible for the successful enhancement. It is highly recommended that, according to the present protocol, anti-CD3ε or anti-CD153 Ab not be used at the isolation step of CD153^+^ SA-T cells, because these Abs may mask CD3ε or CD153 and consequently interfere with the plate-bound Abs at a later stimulation step.

## Resource availability

### Lead contact

Further information and requests for resources and reagents should be directed to and will be fulfilled by the lead contact, Yuji Fukushima (fukushima.yuji.4x@kyoto-u.ac.jp).

### Materials availability

This study did not generate new unique reagents.

## Data Availability

This study did not generate new datasets and code.
